# Probiotics and the Gut Immune System: Indirect Regulation

**DOI:** 10.1007/s12602-017-9322-6

**Published:** 2017-08-31

**Authors:** Giorgio La Fata, Peter Weber, M. Hasan Mohajeri

**Affiliations:** DSM Nutritional Products Ltd., R & D Human Nutrition and Health, P.O. Box 2676, CH-4002 Basel, Switzerland

**Keywords:** Gastrointestinal tract, Microbiota, Probiotic, Immune system, Health status

## Abstract

The gastrointestinal tract (GIT) represents the largest interface between the human organism and the external environment. In the lumen and upper part of the mucus layer, this organ hosts an enormous number of microorganisms whose composition affects the functions of the epithelial barrier and the gut immune system. Consequentially, the microorganisms in the GIT influence the health status of the organism. Probiotics are living microorganisms which, in specific conditions, confer a health benefit to the host. Among others, probiotics have immunomodulatory properties that usually act directly by (a) increasing the activity of macrophages or natural killer cells, (b) modulating the secretion of immunoglobulins or cytokines, or indirectly by (c) enhancing the gut epithelial barrier, (d) altering the mucus secretion, and (e) competitive exclusion of other (pathogenic) bacteria. This review focuses on specific bacteria strains with indirect immunomodulatory properties. Particularly, we describe here the mechanisms through which specific probiotics enhance the gut epithelial barrier and modulate mucus production. Moreover, we describe the antimicrobial properties of specific bacteria strains. Recent data suggest that multiple pathologies are associated with an unbalanced gut microflora (dysbiosis). Although the cause-effect relationship between pathology and gut microflora is not yet well established, consumption of specific probiotics may represent a powerful tool to re-establish gut homeostasis and promote gut health.

## The Microbiota

The human intestinal microbiota is composed of 10^13^ to 10^14^ microorganisms whose collective genome is defined as the microbiome [32, 33, 68]. Along the gastrointestinal tract (GIT), the number of the microorganisms differs enormously and it is mainly represented by bacteria belonging, in order of abundance, to three phyla: Bacteriodetes, Firmicutes, and Proteobacteria [44, 52, 72]. During evolution, the co-existence developed between human and microbiota (referred as symbiotic relationship) has endowed humans with extra-functional features playing a critical role in biological processes such as nutrient utilization, resistance against infections, maturation of the immune system, and host metabolism [4, 5, 9, 11, 16, 54].

The host-microbiota interaction starts already at birth when organisms are believed to be sterile [52, 79]. Recent finding, however, detected microbes in the amniotic fluid, placenta, as well as in meconium and umbilical cord [38, 56, 73]. The diversity of the microbiota is therefore dependent of the mother’s microbiome being partially established at birth and varies further during development. Indeed, a newborn individual will be exposed to different bacteria if delivered by caesarian section or conventionally [22, 54]. Other parameters influencing the microbiota composition include the infant feeding as well as usage of antibiotics early in life [54]. Once established, the host-microbiota interactions are relatively stable during adulthood while decrease in the elderly [8, 33, 56] when chronic and acute perturbations become more frequent and responsible for driving microbiota dysbiosis [9, 78]. Dysbiosis is usually associated with pathological conditions [56] and indicates a state in which the microbiota produce harmful effects to the host via (1) qualitative and quantitative changes in the flora, (2) changes in the metabolic activities of the flora, and (3) changes in the flora distribution [37].

## Probiotics

The importance of keeping a healthy microflora was already clear at the beginning of the twentieth century when consumption of yogurt and a specific mix of bacteria were associated to extended lifespan and prevention of disease [12, 51]. These observations have been the driver for the development of probiotics defined as *“live micro-organisms which, when administered in adequate amounts, confer a health benefit on the host”* [12, 64, 80].

Currently, a large variety of probiotics are available on the market and generally they are consumed to target gastrointestinal discomfort and pain as well as to improve the properties of the immune system. Unfortunately, the health benefits attributed to consumption of specific probiotics are not always fully supported by scientific evidence. It is therefore necessary to invest further in research to describe the mechanisms through which probiotic consumption may influence human health. This concept becomes even more relevant considering the increasing body of evidence associating an altered GIT flora to pathological conditions not directly connected with the GIT like rheumatoid arthritis, ankylosing spondylitis, allergic disorders, autoimmune disease, multiple sclerosis and more recently, psychiatric disorders and memory [36, 37, 42, 53, 64, 66, 80].

## The Microbiota and the Immune System

Being the largest interface between the body and the external environment [20, 28], it is not surprising that the GIT is tightly associated and constantly in communication with the immune system. Intestinal bacteria develop and regulate the host immune system and the immune system affects the composition of the intestinal microbiome [45]. In particular, the host immune system is responsible for ensuring a beneficial microbiota composition controlling specific bacteria overgrowth, but also reacting to pathogenic bacteria or molecules meeting with the intestinal barrier [45]. The interaction between immune system and pathogens is also regulated by microorganisms that can directly interact with pathogenic bacteria or indirectly stimulate the immune system to do the same. Gut homeostasis is therefore reached and maintained when the immune system establishes an appropriate balance between tolerance to commensal (not harmful), mutualistic (beneficial), and opportunistic (pathogenic) bacteria [56]. This balance is consolidated only when the immune system can communicate with the gut microbiota and a key player in this cross-talk process is a healthy intestinal barrier.

In this mini review, we will analyze the interaction between immune system and microbiota. Excellent recent reviews have already addressed the effect that specific bacteria have on the innate and adaptive immune response in human [57, 69, 70]. Therefore, we focus here on three mechanisms by which a selected number of bacteria interact indirectly with the host immune system. The first mechanism focuses on the effects produced by specific probiotic strains on the gut epithelial barrier, the second on mucus secretion and its modulation, and the third explores the antimicrobial properties of specific bacteria (probiotics and not). In this work, when available, the genes and pathways involved in the mechanisms of action are indicated, providing useful information to explore other probiotic properties and potential beneficial effects. To achieve this goal, we first describe briefly the gut intestinal barrier and how its constituents interact with the microbiota and the host immune system.

## The Intestinal Barrier and the Gut Immune System

**Fig. 1:**
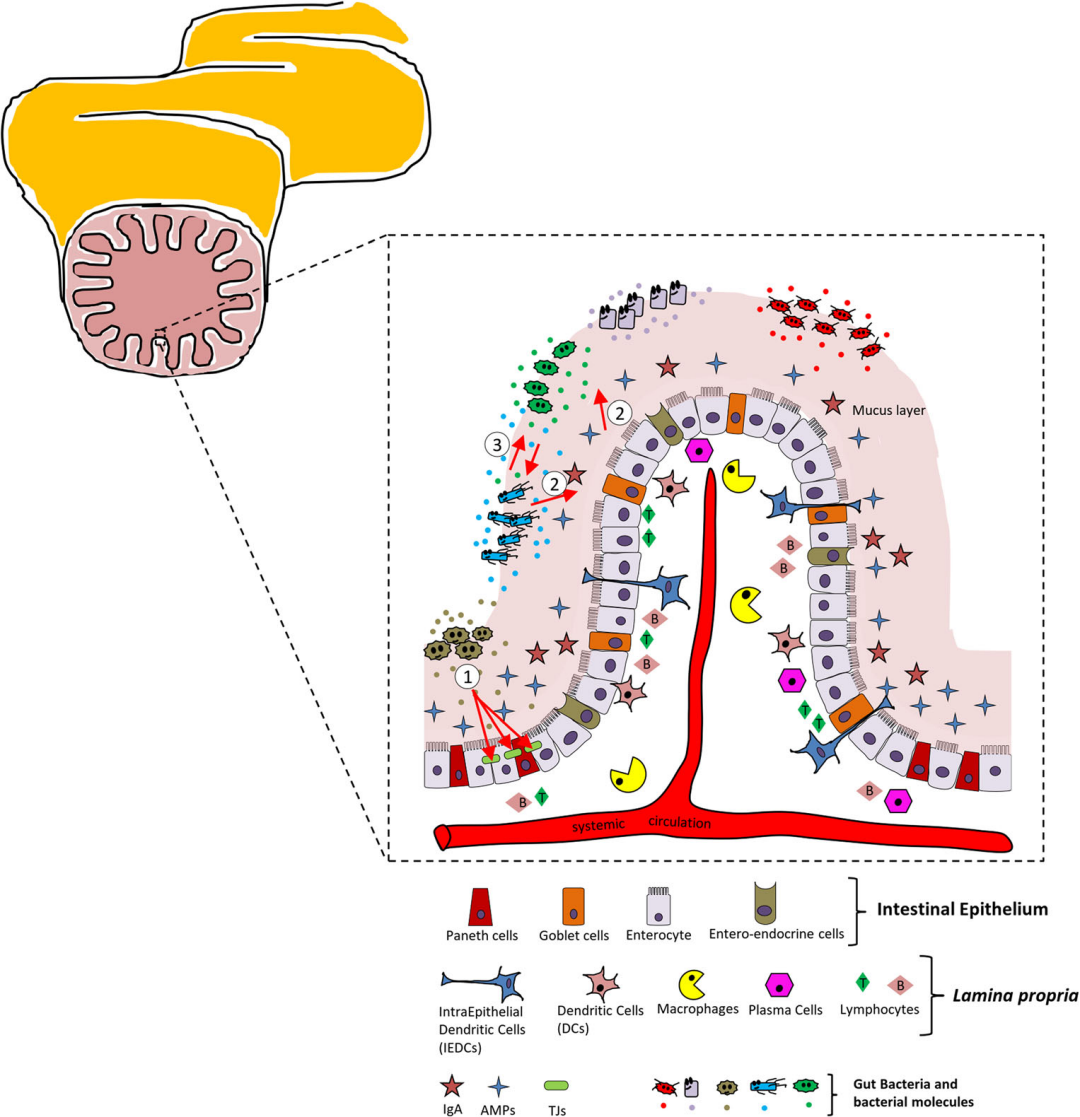
Schematic view of the intestinal barrier and main cellular players. The intestinal tract is presented on the upper right. The enlargement represents a schematic view of the intestinal barrier (mucus layer, intestinal epithelium, and bacterial ecosystems). All different cell types are reported below the enlargement. The red arrows highlight the interactions that specific bacteria strains establish with the intestinal barrier. Outlined here are the ① modulation of the tight junction (TJ) proteins, ② modulation of the mucus secretion, and ③ relationship established between different bacterial populations of the gut ecosystem (antimicrobial properties)

The intestinal barrier is a heterogeneous entity composed of an extra-cellular component: the mucus layer and a cellular compartment including the intestinal epithelium and an underlying *lamina propria* [20] (Fig. 1 and figure legend for details).

Both the mucus layer and the intestinal epithelium, each harboring several specialized cell types, represent a physical barrier to gut microbes. The intestinal epithelium consists of the following: enterocytes (or fluid transporting cells) responsible for absorbing molecules from the intestinal lumen, Paneth cells specialized in synthetizing and secreting antimicrobial peptides (AMPs) upon contact with enteric bacteria, and mucus-secreting Goblet cells and entero-endocrine cells [20, 44, 76]. In humans, the intestinal epithelium is renewed every three to 5 days, a fact that, per se, is immune protective as it removes infected or damaged cells [10]. Moreover, the permeability of the GIT epithelium has also immune protective activities and it is under the control of the tight junction (TJ) proteins. The expression of these proteins has been demonstrated to be regulated by specific probiotics and therefore TJs are discussed more in detail later in this text.

The *lamina propria* represents the interface between host and immune system in the gut and consists of dendritic cells, macrophages, and plasma cells, in addition to B and T lymphocytes.

Macrophages are strategically positioned in the sub-epithelial regions where antigens may cross the intestinal epithelium and are specialized in phagocytosing potentially harmful microbes as well as scavenging apoptotic cells and debris [40, 69]. Intestinal dendritic cells phagocytose apoptotic cells, sample bacteria in the mucus, and subsequently, migrate to the lymph nodes where they activate T cells and the inflammatory response [69]. Plasma cells and T cells regulate the humoral response of the GIT via secretion of immunoglobulin (IgA) and several cytokines and inflammatory mediators [69].

## Mechanisms of Action

### Probiotics and the Tight Junctions in the Gut Epithelium

One of the functions of the GIT epithelium is to establish a physical barrier between external environment and host immune system. Therefore, the functionality and integrity of this barrier are keys to support the permeability to nutrients and beneficial molecules albeit protecting the host from dangerous threats.

**Fig. 2:**
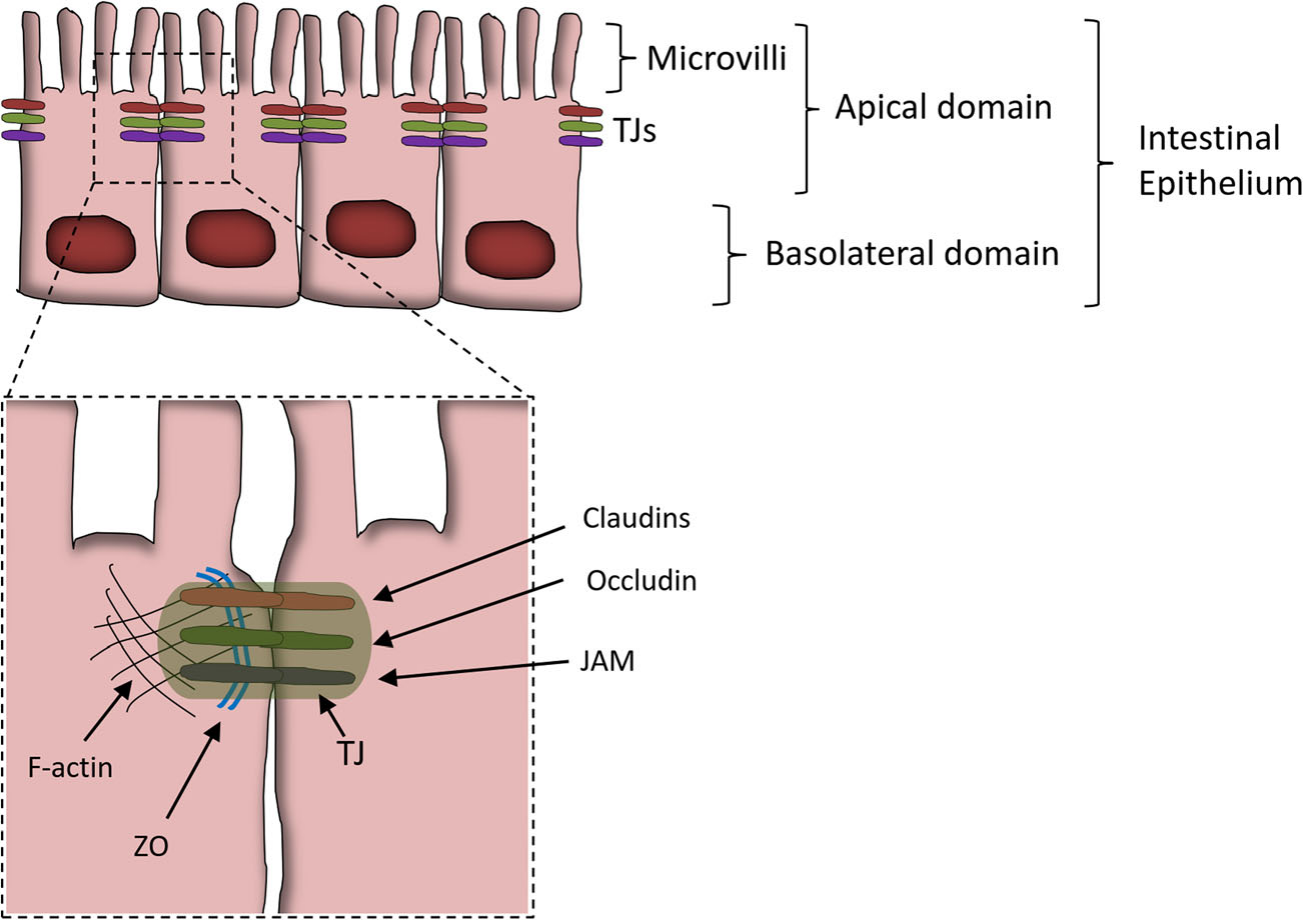
Schematic view of the gut epithelial tight junctions (TJs). Schematic and simplified view of the GIT epithelium. The dashed rectangle is enlarged below and indicates more details of the TJ structure. The proteins constituting the TJ are indicated. Abbreviations: F-actin (filamentous-actin), ZO (zonula occludens) 1-2-3, JAM (junctional adhesion molecule). Other proteins constituting the TJ and not represented in the figure include the following: myosin II (part of the cellular cytoskeleton) and tricellulin (at junction between three cells)

The integrity of the GIT epithelium is partially guaranteed by multi-protein complexes defined as TJs (Fig. 2) [29, 43, 75].

TJ’s function and biology have been extensively reviewed elsewhere (for details, refers to [75] and references within). In brief, TJs are located in the apical part of the intestinal epithelial cells. They consist of transmembrane proteins that extracellularly interact with similar domain of neighboring TJs and intracellularly connect with the cellular cytoskeleton (Fig. 2).

When the expression or localization of the TJ proteins is altered, the functionality of this physical barrier is compromised and the leaky gut condition may develop [75].

The leaky gut is characterized by having an epithelium with increased permeability to molecules/compounds that diffuse from the lumen to the *lamina propria*. This condition can be measured in vitro by the trans-epithelial electrical resistance (TEER) parameter, or in vivo by the intestinal permeability test (IPT) [50]. Leaky gut is responsible for the development of multiple pathological conditions such as irritable bowel disease (IBD), irritable bowel syndrome (IBS), and celiac disease [41], all characterized by sustained inflammation and tissue damage.

Multiple compounds in the diet have already been demonstrated to regulate the expression of the TJ proteins [20] and similarly, probiotics also may regulate the expression/localization of these proteins (Table 1).

**Table 1:** List of probiotic strains improving the intestinal epithelium properties via TJ modulation

Bacterial strain	Mechanism of action	Increased(↑) or decreased(↓) gene/protein expression	Reference
*Escherichia coli* Nissle 1917	- EcN inhibits the leaky gut condition by upregulation of the zonula occludens-1 (ZO-1) in murine intestinal epithelial cells	↑ZO-1	[74, 83]
	- EcN protects against the increased mucosal permeability in the dextrane sodium sulfate (DSS)-induced colitis murine model	↑ZO-2	
	- T84 monolayer cells in vitro treated with EcN showed increased ZO-2 expression and ZO-2 redistribution (concentration at the sites of cellular contacts). The redestribution of ZO-2 seems to be regulated (in part) by activity of the protein kinase C-zeta (PKC-ζ)	For both: gene and protein expression	
*Lactobacillus rhamnosus* GG	In vitro pretreatment of MDCK-I and T84 epithelial cell monolayers with *L. rhamnosus* GG prevents injuries induced by enterohemorragic *Escherichia Coli* O157:H7 infections via regulation of ZO-1 (protein expression and distribution) and Claudin-1 (distribution)	↑ZO-1 Protein expression	[39]
		Claudin-1 Protein redistribution only	
*Lactobacillus casei* DN-114001	- In vitro T84 epithelial cell monolayer treated with L. casei are protected against the enteropathogenic *Escherichia Coli* E2348/69	ZO-1 Protein redistribution only	[55]
	- *L. casei* inhibits the redistribution of ZO-1 following the infection with *E. coli*		
*Streptococcus thermophilus* ATCC19258 *Lactobacillus acidophilus* ATCC4356	Probiotic pretreatment of the human intestinal epithelial cell lines HT29/cl.19A and Caco-2 exposed to *Escherichia coli 029:NM* maintained the phosphorylation levels of ZO-1, occludin and actinin	ZO-1 Occludin Actinin For all: phosphorylation status only	[62]
*Bifidobacterium infantis* (From VSL#3 cocktail)	T84 cell lines treated with bioactives released in the medium by *B. infantis* decreased claudin-2 and phospo-p38 (p-p38) expression, and increased ZO-1, occludin and phospo-ERK (P-ERK) expression	↑ZO-1 ↑Occludin ↑Claudin-4 ↓Claudin-2 ↓P-p38 ↑P-ERK For all: protein expression only	[27]
*Lactobacillus plantarum MB452*	Caco-2 cells treated with *L. plantarum* showed increased gene and protein expression of occludin, ZO-1, ZO-2, cingulin, itchy homolog E3 ubiquitin protein ligase (ITCH), snail homolog 1 (SNAI1), and others (reference for complete list)	↑Occludin ↑ZO-1 ↑ZO-2 ↑Cingulin ↑ITCH ↑SNAI1	[1]
*Lactobacillus plantarum* WCFS1	- Administration of *L. plantarum* directly in the duodenum of human healthy volunteers showed increased ZO-1 and occludin proteins	↑ZO-1 ↑Occludin TLR-2 (activation)	[41]
	- In in vitro Caco-2 model *L. plantarum* was shown to activate the Toll-like receptor (TLR)-2 signaling pathway		
*Lactobacillus plantarum* CGMCC No.1258	*L. plantarum* protected the integrity of Caco-2 monolayer cells against damages by entero-invasive Escherichia coli via TJ proteins regulation (expression and distribution)	↑Claudin-1 ↑Occludin ↑JAM-1 ↑ZO-1	[58]

To study the effect of *Escherichia coli* Nissle 1917 (EcN) in vivo, healthy germ-free mice were colonized with this probiotic and the gene expression in their intestinal epithelial cells (IECs) was analyzed [74]. Colonization of these mice with EcN resulted in an up-regulation of ZO-1 in IECs at both mRNA and protein levels [74]. Similar data were also observed when EcN was administered to murine models for colitis suggesting that upregulation of the ZO-1 stabilizes the TJs and therefore improves the barrier function of the intestinal epithelium [74]. To investigate the molecular mechanism by which EcN contributes to the gut barrier integrity, Zyreck and collaborators used the T84 monolayer cells as in vitro model [83]. Although, in this case, they did not detect differences in ZO-1 expression, DNA microarray identified ZO-2 as key gene responsible for the probiotic effect associated to EcN [83]. Indeed, EcN stimulated the over-expression of the ZO-2 and redistribution (regulated partly via PKC-zeta activity) of this protein to the site where cellular contacts occur, to stabilize the TJs and maintain cell morphology [83].

The properties of the intestinal epithelium are also influenced by another probiotic. *Lactobacillus rhamnosus* strain GG and its protective effects against the enterohemorragic *Escherichia Coli* O157:H7 infections were demonstrated in vitro in MDCK-I and T84 epithelial cell monolayers [39]. Specifically, Johnson-Henry and collaborators showed that the epithelial cells treated with the probiotic prior to *E. coli* infection maintained higher levels of ZO-1 expression than those infected with the pathogen alone [39]. Similarly, the distribution of the claudin-1 protein was also retained when the cells were pretreated with the probiotic *L. rhamnosus* GG [39]. In this case, the stability of the epithelial barrier structure was guaranteed by ZO-1 and claudin-1, both important TJ proteins.

Similarly to *L. rhamnosus* GG, another probiotic strain has a protective effect against pathogen infections via regulation of TJ proteins. In the T84 epithelial cells monolayer model, *Lactobacillus casei* DN-114001 stabilizes the ZO-1 distribution against the enteropatogenic Escherichia coli E2348/69 [55].

*Lactobacillus plantarum* MB452 upregulates the gene and protein expression of ZO-1, ZO-2, occludin, and cingulin [1], as well as the expression of other genes involved in the degradation of TJ proteins such as ITCH and SNAI 1 [1] and Table 1). An increased expression of the ZO-1 and occludin genes was also observed for *Lactobacillus plantarum* strain WCFS1 [41] and *Lactobacillus plantarum* strain CGMCC No.1258 [58]. Specifically, Karczewski and collaborators administered *Lactobacillus plantarum* strain WCFS1 directly in the duodenum of healthy subjects by a feeding catheter. They measured the intestinal barrier parameters in the human tissue and suggested that the activation of the TLR2-dependent pathway is responsible for regulating the expression and distribution of the TJ proteins [41].

Of note, there are probiotic strains modulating TJs functions by altering the phosphorylation status of the TJ proteins only, without altering their gene expression. Pretreatment of the human intestinal epithelial cell lines HT29 and Caco-2 with *Streptococcus thermophilus* ATCC19258 and *Lactobacillus acidophilus* ATCC4356 for example, maintained the phosphorylation levels of ZO-1, occludin, and actinin when the cellular models were exposed to infections by *Escherichia coli* 029:NM [62].

### Probiotics Modulating the Properties of the Mucus Layer

The intestinal epithelium is covered by a viscoelastic mucus layer whose main functions are to (a) build a protective barrier against the harsh luminal environment (containing digestive enzymes), (b) facilitate food passage, and (c) avoid firm adhesion of bacteria to the epithelial cells thus preventing their entry into the *lamina propria* [17, 20, 21]. By limiting the interaction and penetration of bacteria, a healthy mucus layer plays an important role in preventing inflammatory and infectious diseases. The mucus in the GIT is produced by Globet cells residing in the intestinal epithelium and it is mainly composed by mucins. Mucins are high molecular weight glycoproteins divided in two groups: secreted mucins (coded by the MUC2, MUC5AC, MUC5B, and MUC6 genes) that are responsible for the formation of the mucus layer and transmembrane mucins (MUC1, MUC4, MUC13, MUC16) whose function is still poorly understood but likely involved in signaling pathways [17, 18, 47]. Among the different human mucin genes, MUC2 and MUC3 are the ones predominant in the colon [47]. Altered expression of specific mucins was associated to gastrointestinal diseases such as Crohn’s disease [14] and ulcerative colitis [63] highlighting the importance of these proteins in the GIT.

Specific probiotic bacterial strains have been demonstrated to regulate mucin expression therefore influencing the properties of the mucus layer and indirectly regulate the gut immune system. A list of these probiotics is reported in Table 2 and their mechanism of action is reported more in detail below.

**Table 2:** List of probiotic strains regulating the mucus layer

Bacterial strain	Mechanism of action	Increased (↑) or decreased (↓) gene/protein expression	Reference
*Lactobacillus plantarum* 299v	In vitro (HT-29 cell lines)*,* Lp299v reduces the adherence of enterophatogenic *Escherichia coli* to mucosal epithelial cells via increasing the expression of mucins 2 and 3 at mRNA level	↑MUC2 ↑MUC3 For both: gene expression	[36, 47]
*Escherichia coli* Nissle 1917	In vitro incubation of HT-29 cells with EcN increases the expression of multiple mucin genes. Milder effects were observed using inactivated bacteria while stronger effects were shown for polarized cells	↑MUC2 ↑MUC3 ↑MUC5AC ↑MUC5A For all: gene and protein expression	[35]
*Lactobacillus casei* GG	In vitro addition of LGG to Caco-2 cells reduces *Escherichia coli* translocation via increased expression of MUC2 gene expression	↑MUC2 Gene and protein	[49]
*VSL#3* (probiotic mixture)	In vivo and in vitro experiments show that exposition to VSL#3 increases the gene expression levels of MUC2 and only mildly of MUC1 and MUC3	↑MUC2 ↑MUC1 ↑MUC3 Gene expression	[15]

*Lactobacillus plantarum* strain 299v was demonstrated to inhibit the adherence of the enteropathogenic *Escherichia coli* to the intestinal epithelial HT-29 cell line [47]. Incubation of *L. plantarum* strain 299v with HT-29 increased the mRNA expression of the MUC2 and MUC3 genes, suggesting that this probiotic induces epithelial cells to secrete mucins that diminish enteric pathogens binding to mucosal epithelial cells [47]. These results are in agreement with previous studies performed by Bernet and collaborators who observed similar effects using *Lactobacillus acidophilus* strain LA1 in Caco-2 cells [7, 47].

The probiotic *Escherichia coli* Nissle 1917 was also demonstrated to alter the expression of mucin genes [35]. Specifically, incubation of HT-29 cells with *E. coli* Nissle 1917 showed an increased expression of MUC2, MUC3, MUC5AC, and MUC5A genes. This effect was stronger when a basal stimulation model was used and, with exception to MUC3, it was not observed when the only bacteria medium was used [35]. A possible explanation provided by the author regards the localization of the Toll-like receptors (TLRs) which are more abundantly present in the basal surface of the cells [34, 35] and represent key signaling regulators of the immune response [34].

Another probiotic able to regulate mucin expression is the *Lactobacillus casei* strain GG. In multiple in vitro models, it was shown that *L. casei* GG inhibited the translocation of specific pathogenic bacteria adhering to the receptors of cultured enterocytes [49] and references within) via for example, an up-regulation of the MUC2 gene expression [49].

Further evidences supporting the above-mentioned effects were also obtained by other in vitro and in vivo studies. Caballero-Franco and collaborators indeed administered a probiotic formula (VSL#3) to rat models and observed a 60% increase in the basal mucin luminal content [15]. Subsequently they demonstrated that VSL#3 was inducing a significant over-expression of the MUC2 gene as well as a similar, although milder, effect for MUC1 and MUC3 genes in vitro [15]. In this case, however, the specific contribution of each bacterial strain could not be determined.

### Bacteria with Antimicrobial Properties

Specific bacteria strains have been described to have antimicrobial properties usually associated with secretion of peptides or molecules which enables them to compete within the complex gut ecosystems. These molecules may protect the host against infectious bacteria and favor the survival of commensal bacteria [13]. A list of bacteria with antimicrobial properties is presented in Table 3.

**Table 3:** List of bacterial strains with proven antimicrobial properties

Bacterial strain	Antimicrobial effect	Reference
*Lactobacillus brevis* 925A	Brevicin 925A has antimicrobial effect against *Listeria monocytogenes* and *Streptococcus mutans*	[77]
Lactobacillus fermentum CS57	In vitro antimicrobial activity against *Streptococcus agalactiae* and *Candida albicans* (vaginal ecosystem) Bacteriocin-like substance as antimicrobial molecule	[67]
*Lactobacillus johnsonii* NCC 533 (previously Lactobacillus acidophilus LA1)	Spent culture supernatant from LA1 contains antimicrobial components that reduce the amount on *Salmonella typhimurium* in vivo (murine models of infections) and in vitro (Caco-2 model)	[6, 44]
	Antimicrobial activity by LjNCC533 associated to lactic acid and hydrogen peroxide production	[2]
*Lactobacillus salivarius* UCC118 and DPC6488	Secretion of the Abp118 bacteriocin with antimicrobial activity against the *Listeria monocytogenes* in vivo model (rodents) *Lactobacillus salivarius* DPC6488: secretion of two bacteriocins (salivaricin L and T) with analogies with Abp118 (see reference for details)	[19, 24]
*Lactobacillus plantarum* G1 and G3	Bacteriocin-like activity identified and characterized with antimicrobial properties (for specificity see reference) Potential probiotics: high vitality in the GIT, no toxicity (safety), improved lipid metabolism, and hepatic function	[82]

*Lactobacillus brevis* strain 925A influences the gut immune system via the production of a bacteriocin identified as brevicin 925A. Brevicin 925A was found to be effective against *Listeria monocytogenes* and *Streptococcus mutans* which cause food poisoning and dental caries [77]. Although the functional analysis of the gene coding for this compounds was not yet completed, similar bacteriocin compounds were also identified in other bacteria strain: *Lactobacillus plantarum* strain TMW1.25 [25, 61, 77].

*Lactobacillus fermentum* strain CS57 was recently isolated from vaginal swabs and shown to produce a bacteriocin-like substance (BLS), with a wide spectrum of antimicrobial activity [67]. With a molecular weight greater than 30 kDa, the BLS was identified as probably belonging to class III bacteriocins, i. e., heat-labile bacteriocin, whose coding genes are not yet identified. Functionally, the BLS produced by *L. fermentum* CS57 demonstrated a strong in vitro antimicrobial activity against *Candida albicans* and *Streptococcus agalactiae* responsible of serious infections when newborns pass through the cervical canal [67]. Similar effects were also observed earlier by combination of BLS produced by two other Lactobacilli: *Lactobacillus rhamnosus L60* and *Lactobacillus fermentum L23* [65]. Taken together, these data suggest that these strains may be used as potential probiotics against vaginal infections and in favor of a healthy vaginal ecosystem [65, 67].

*Lactobacillus johnsonii* NCC 533 was daily administered to mice infected with *Salmonella typhymurium.* Fecal analysis showed that the *S. typhymurium* was reduced even after the administration was stopped suggesting that LA1 is able to survive in the intestines [6]. Although the mechanism of action was not described, the authors suggested that it may involve stimulation of the immunological defenses or secretion of antimicrobial compounds [6].

*Lactobacillus salivarius* UCC118 is a well-characterized bacterial strain secreting a potent broad spectrum of small heat-stable proteins belonging to class II bacteriocins [19]. Using infected mice models, Corr and collaborators demonstrated that oral administration of *L. salivarius* UCC118 was sufficient to reduce the infection of *Listeria monocytogenes* particularly in the liver and spleen [19]. This effect was attributed to the secretion of the bacteriocin Abp118 acting directly on the target cell and not via intermediate mechanisms [19]. Of note, in a microarray-based comparative genome hybridization analyses, Eileen and collaborators identified two novel bacteriocins in *L. salivarius* DPC6488 with analogies to the Abp118: salivaricin L and T [24].

*Lactobacillus plantarum* G1 and G3 were identified by Zavisic and collaborators [82]. The antimicrobial and bacteriocin properties of these strains were specific for multiple pathogenic bacteria such as *Staphylococcus aureus*, *Escherichia coli*, and *Salmonella abony* [82]. In addition to the antimicrobial properties of this strain, the authors described also high degree of viability in the gastrointestinal tract, absence of toxicity following high dose of oral administration (mice), as well as improved lipid metabolism and hepatic function (rats). Therefore, the authors propose *Lactobacillus plantarum* G1 (and G3) as potential novel probiotics [82].

## Discussion

The physiological equilibrium established between microorganisms colonizing human intestinal tract and host is key to the health status of each individual. This equilibrium relies on complex and dynamic relationships within bacterial ecosystems and host immune system. In this manuscript, three mechanisms through which specific bacteria strains indirectly communicate with the immune system have been described. The first explores the effect that probiotic have on the gut epithelial barrier and in particular the tight junctions, the second focuses on bacteria that by communicating with the intestinal epithelium may alter the properties of the mucus layer, while the third describes the antimicrobial molecules that specific bacteria strains use to compete within the gut ecosystems.

The GIT epithelium represents a physical barrier between external environment and host immune system. The integrity of such barrier is regulated by multi-protein structures called tight junctions that are key to regulate the gut permeability to nutrients and beneficial molecules while protecting the host from threats originating within the GIT.

Multiple probiotics modulate the expression of proteins constituting the TJs and therefore were reported in this work. The studies indicated here highlighted the importance of proteins like ZO-1 and occludin whose expression is regulated by specific bacteria such as *Escherichia coli* Nissle 1917, *Lactobacillus rhamnosus* strain GG, and *Lactobacillus casei* strain DN-114001 [39, 55, 74, 83]. In general, while most of the studies describe that probiotics modulate the expression and distribution of these and other proteins in monolayer cells in vitro, few reports describe a regulation of their phosphorylation status [27, 62]. Both expression levels and phosphorylation status of the proteins constituting the TJ structure are key mechanisms by which known probiotics or potential new ones may alter the fine equilibrium of barrier and permeability assured by the gut epithelium.

The mucus is a complex viscous proteinaceous continuous layer in the gut lumen representing the first line of defense of the host against environmental threats [21]. It is a highly hydrated gel containing glycoproteins like mucins and other important constituents such as defensins, immunoglobulins, and trophic factors [21]. Specific studies reported in this work have provided valuable information about the mechanisms by which certain bacteria strains regulate the gene expression of mucins and therefore affect the properties of the mucus layer. Such studies relied on the HT-29 and Caco-2 in vitro cellular models. Pre-incubation of HT-29 cell lines with *Lactobacillus plantarum* 299v resulted in a reduced adherence of the enteropathogenic bacteria *Escherichia coli* to the intestinal cells [47]. In this case, the effect was driven by an increased expression of the MUC2 and MUC3 genes. The mechanisms driving the over-expression of MUC2 and MUC3 were investigated further and suggested that both, a direct contact of bacterial cells with intestinal cells as well as indirect stimulation of intestinal cells via secreted molecules, were involved.

Moreover, Dykstra and collaborators showed that the interaction of this probiotic strain with the cells was responsible to enhance the cellular-driven protection via reduction of the caspase pathway activation [23]. The importance of mucins was also highlighted by other observations demonstrating a reduced number of Goblet cells in inflammatory lesions of the GIT as well as a decreased functional capacity of the mucins to bind pro-inflammatory molecules and to inhibit bacterial binding in the inflamed colon [30, 46, 47]. Similar data were also observed previously using Caco-2 cell line and the probiotic strain *Lactobacillus acidophilus LA1* [7, 47]. These data are in agreement with the effects observed years later for *Escherichia coli* Nissle 1917 where, in addition to MUC2 and MUC3, other genes were also affected such as MUC5AC and MUC5A [35]. In this case, however, except for MUC3, the cultured medium did not affect the mRNA levels of the other genes.

These data not only highlight the importance of in vitro models to determine the mechanisms through which common and potential probiotics may confer health benefit to the host, but also argue that different bacterial strains have strain-specific effects that cannot be extended to other bacterial species.

The limitations associated with using in vitro studies are represented by absence of complementary effect that two or more species have influencing each other as well as absence of the bacteria-host interaction effects. In this case, a more powerful source of information can derive by in vivo models. In a recent animal study for example, *Bactaeriodes thetaiotaomicron* was demonstrated to increase Goblet cell differentiation and expression of mucus-related gene favoring mucus production. This effect was diminished when *B. thetaiotaomicron* was associated with *Faecalibacterium prausnitzii*. This study reveals the importance of the balance between metabolically complementary commensal bacteria in maintaining colonic epithelial homeostasis [81].

Oral administration of *Lactobacillus acidophilus* LA1 was shown to be protective against *Salmonella typhimurium* infections [6]. Of interest, a combination of in vitro and in vivo experiments suggested that the antimicrobial properties associated with LA1 were specific for *S. typhimurium* and associated with secreted molecules released by this bacterial strain [6]. Bacteriocins belong to this class of molecules and received lots of attention in recent years for their potential to be used as alternative therapies to antibiotics or in the preservative industry. For example, *Bacillus thuringiensis*, a bacterium isolated from human feces, produces the bacteriocin thuricin CD [60]. This bacteriocin was shown to exhibit antibacterial properties activity against *C. difficile* as well as *Listeria monocytogenes* without affecting other constituents of the GIT microbiota [60]. Of note, other molecules with microbicidal properties exist and they can be released by cells of the intestinal barrier directly upon stimulation by specific bacteria. This is, for example, the case of the alpha-defensin peptides released by Paneth cells in response to bacteria stimulation, contributing therefore to the innate immune response of the GIT [3]. Interestingly, the release of these molecules was regulated by bacteria only (in this case: *Salmonella typhimurium, Escherichia coli* and *Staphylococcus aureus*) and not by other microorganisms such as fungi or protozoa [3].

Other examples include the *Lactobacillus plantarum* G1 and G3 whose beneficial effects were measured by improved lipid metabolism and improved hepatic function in Wistar rats [82].

Of note, although animal models represent excellent tools to investigate the microbiome-host interactions and possible beneficial effects in vivo, they lack of the “simplicity” to establish the mechanisms of actions driving such benefits. Therefore, a combination of in vitro and in vivo models is essential to investigate the mechanisms of action of known probiotics as well as of novel potential probiotics.

This is the case for *F. prausnitzii*, an abundant anaerobic bacterium present in the human gut and belonging to the Clostridium leptum phylogenic group (Firmicutes) [59, 71]. Multiple studies have reported that *F. prausnitzii* is depleted in the mucosa of patients with inflammatory bowel disease (IBD) [31, 48, 71] suggesting that this bacterium has a role in the IBD prevention. Indeed, *F. prausnitzii* was demonstrated to have anti-inflammatory properties both in vitro (in peripheral blood mononuclear cells (PBMCs) and Caco-2 cells) as well as in trinitrobenzenesulfonic acid-induced colitis animal models in vivo [71] possibly through the secretion of specific metabolites that would control the inflammatory pathway [59, 71].

Another bacterium with potential probiotic properties is *Akkermansia muciniphyla,* a mucin-degrading bacterium that resides in the mucus layer and whose presence is inversely correlated with body weight in rodents and humans [21, 26]. Also in this case, using a mouse model for type 2 diabetes, the authors describe multiple beneficial effects associated with administration of *A. muciniphyla* including control of inflammation, gut barrier, and gut peptide secretion mediated by endocannabinoids regulation [26].

## Conclusions

In conclusion, modulation of the immune response associated with consumption of specific probiotics may occur not only via the innate and adaptive immune system, but also via (a) regulation of the intestinal epithelium permeability, (b) mucus secretion, and (c) competition within bacterial ecosystem via secretion of antimicrobial compounds. These mechanisms can be easily assessed in in vitro setting and therefore represent valid tools to study the properties of newly discovered bacteria strains.
